# 非小细胞肺癌*EGFR*基因异质性研究进展

**DOI:** 10.3779/j.issn.1009-3419.2011.11.11

**Published:** 2011-11-20

**Authors:** 华 郑, 红梅 张, 鹤玲 史, 宝兰 李

**Affiliations:** 101149 北京，首都医科大学附属北京胸科医院综合科 General Department, Beijing Chest Hospital of Capital Medical University, Beijing 101149, China

表皮生长因子受体酪氨酸激酶抑制剂（epidermal growth factor receptor-tyrosine kinase inhibitors, EGFR-TKIs）分子靶向治疗的问世使晚期非小细胞肺癌（non-small cell lung cancer, NSCLC）的治疗获得了突破性进展。它以疗效明显、毒副反应轻、口服方便等优点迅速占据了肺癌市场。日本和韩国^[1, 2]^分别报道了吉非替尼上市前后晚期NSCLC患者生存的回顾性研究，结果发现吉非替尼上市后患者的生存期较上市前明显延长。研究^[3]^发现TKI的疗效与EGFR的突变状态密切相关，EGFR的突变主要发生在外显子18-21，缺失突变主要发生在外显子19，最常见的是del E746-A750，替代突变最常见的是发生在外显子21上的L858R，这两处突变均为激活突变，即它们能导致不依赖于配体的EGFR酪氨酸激酶的激活，约占突变的90%^[4]^，另外还有包括外显子20的T790M的耐药突变，可导致原发或继发TKI耐药^[5]^。但是在*EGFR*基因突变阳性的患者之间TKI（吉非替尼或厄洛替尼）的疗效却存在很大差异，最近研究提示这种差异可能与*EGFR*基因的异质性有关。*EGFR*突变异质性是一个非常新的概念，同一病灶的不同部分、同一患者的不同病灶、甚至同一病灶治疗前后*EGFR*突变状态都可能不一致^[6]^。发生*EGFR*基因异质性的原因可能是肿瘤细胞在转移过程中发生新的突变^[7]^，或者在原发灶发生*EGFR*突变时就已经形成了异质性^[8]^。本文旨在对这方面的研究结果进行综述。

## 1 同一肿瘤组织内*EGFR*基因异质性

Taniguchi等^[9]^对21例具有*EGFR*突变、术后复发后仅接受了吉非替尼治疗的NSCLC患者标本进行分析，利用显微切割技术，对每一例肿瘤标本选取50个-60个瘤灶进行*EGFR*基因突变检测。21例患者中15例全部由*EGFR*突变细胞组成，但其它6例由部分突变和部分未突变的细胞组成，其中17例靶向治疗有效（81%）。6例由部分突变和部分未突变的细胞组成的患者的无疾病进展生存期（progression free survival, PFS）和总生存期（overall survival, OS）均明显少于全部突变者（*P*=0.009, *P*=0.003），表明肿瘤组织内*EGFR*突变的异质性是影响靶向治疗疗效的重要因素。

Tomonaga等^[10]^最近报道了混合型肺腺癌肿瘤组织内的异质性研究。该项研究测定了38例混合型肺腺癌，每例纤维微切割的样本均由病理学家将其分为乳头状型、腺泡型和肺泡细胞型等亚型。结果在9例组织中检测到肿瘤内EGFR异质性，而且*EGFR*突变更常见于肺泡细胞型，但无统计学差异; *EGFR*突变肿瘤内异质性与吸烟史有关（*P* < 0.043）。结果提示在腺癌中EGFR的异质性可能与不同类型的细胞组成有关系。

上述研究提示肿瘤组织内部普遍存在EGFR异质性，而且这种异质性会影响TKI治疗的疗效。

## 2 同一患者原发灶与转移灶*EGFR*基因异质性

研究证实原发灶与转移灶之间也存在*EGFR*基因异质性。Gow等^[11]^分析了67例从未接受过TKI的NSCLC配对原发灶和转移灶的EGFR外显子18-21的检测结果。转移灶包括脑、骨、胸膜、皮肤、软组织、淋巴结、消化系统、肺、肾上腺等。用直接测序法，18例具有原发灶*EGFR*突变的患者中9例转移灶丢失突变，26例转移灶中存在突变的患者中17例原发灶是阴性的。用扩增阻滞突变系统法（scorpion amplified refractory mutation system, SARMS）也测定了上述标本。结合两种方法原发灶和转移灶的不一致率为27%（18/67），提示原发灶与远处转移灶之间存在*EGFR*基因异质性。

Schmid等^[12]^用直接双向测序法测定96例配对的肺腺癌原发灶和局部淋巴结转移灶中*EGFR*基因外显子18-21突变状态。7例发现*EGFR*基因突变，其中1例原发灶和淋巴结转移灶中的*EGFR*突变是一致的。3例是原发灶有突变而转移灶没有，另3例是原发灶没有而转移灶有，不一致率为6.25%（6/96），结果提示在原发灶与局部转移淋巴结之间也存在*EGFR*基因异质性。

原发灶与转移灶的异质性也是影响TKI疗效的因素。Sun等^[13]^用直接测序法测定80例NSCLC患者的原发灶和区域转移淋巴结中*EGFR*基因突变。160例标本中21例原发灶和26例转移灶显示*EGFR*突变。*EGFR*突变在原发灶和转移灶的不一致率为8.75%（7/80）。结果提示在中国有相当比例的NSCLC患者显示*EGFR*基因突变在原发灶和转移灶中不一致。其中，5例纵隔淋巴结具有EGFR敏感突变的患者接受了吉非替尼新辅助化疗，3例为外显子19缺失突变，2例为外显子21错义突变，结果显示4例对吉非替尼治疗有效，1例显示进展。有效的4例中原发灶和转移灶均缩小，追溯原发灶的*EGFR*突变时发现，4例均具有*EGFR*敏感突变，进展的1例显示原发灶增大和出现胸腔积液，进一步取原发灶进行基因检测发现原发灶的EGFR为野生型，提示原发灶和转移灶的*EGFR*基因异质性是影响TKI疗效的重要因素。

还有很多其它的研究结果（表 1），不同实验室原发灶与转移灶的*EGFR*基因突变不一致率存在一定的差异，可能与患者入选标准不同以及检测方法不同有关，但是均提示NSCLC中*EGFR*基因在原发灶和转移灶中确实存在不同程度的异质性。

**1:** 原发灶与转移灶中*EGFR*基因突变检测不一致率的比较 Comparison of *EGFR* mutation between primary and metastatic tumors

Author	No. of specimen	Detection technology^a^	Metastatic tumor^b^	Discordant rate (%)
Gow^[11]^	67	Sequencing SARMS	Br, Bo, Ly, GI, Lu, AG, Pl, Sk, ST	26.9% (18/67)
Schimid^[12]^	96	Sequencing	Ly	6.3% (6/96)
Sun^[13]^	80	Sequencing	Ly	8.8% (7/80)
Kalikaki^[7]^	25	Sequencing	Sk, Lu, TW, Br, AG, Li, Bo	28.0% (7/25)
Matsumoto^[14]^	19	Sequencing	Br	0 (0/6)
Park^[15]^	101	Sequencing Heteroduplex analysis	Ly	11.9% (12/101) 16.8% (17/101)
Gomez-Roca^[16]^	49	IHC	-	32.7% (16/49)
Badalian^[17]^	11	IHC	Bo	54.5% (6/11)
Rao^[18]^	51	IHC	Ly	10.6% (5/47)^c^ 19.2% (9/47)
Watzka^[19]^	39	IHC	-	30.8% (12/39)
Italiano^[20]^	30	IHC FISH	AG, Bo, Br, Lu, ST	33.3% (10/30) 26.9% (7/26)
Bozzetti^[21]^	31	FISH	Li, Pl, Ab, Ri, Sk, Ly	32.1%（9/28）
Daniele^[22]^	28	FISH	Br, AG	22.9% (8/35)
Monaco^[23]^	40/366	FISH	Ly, Pl, Br, PF, Li, Bre	32.5% (11/34)
Fang^[24]^	35	Taqman RT-PCR	Ly, Br	31.5% (11/35)
^a^IHC: immunohistochemistry; FISH: fluorescence in situ hybridization; ^b^Ab: abdomen; AG: adrenal gland; Br: Brain; Bre: breast; Bo: bone; GI: gastrointestinal system; Li: liver; Lu: metastatic lung tumor; Ly: lymph node; PF: pleural fluid; Pl: pleura; Ri: ribs; Sk: skin; ST: soft tissue; TW: thoracic wall; ^c^Classification method is different between upper row and lower row. Upper row: (-/+, ++, +++), Lower row: (-, +/++, +++).				

## 3 肿瘤组织与血液的*EGFR*基因异质性

肿瘤组织与血液的DNA *EGFR*基因突变状态也存在差异。Bai等^[25]^利用高压液相色谱法（denaturing high-performance liquid chromatography, DHPLC）分析了230例Ⅲb期和Ⅳ期NSCLC患者血浆DNA样本和对应肿瘤组织的*EGFR*基因外显子19和21突变。230例标本中79例血浆*EGFR*突变，77例肿瘤组织中*EGFR*突变，16例血浆DNA突变的患者没有对应原发灶的突变，14例肿瘤组织EGFR突变的患者血浆DNA未检测到突变，不一致率为13.0%（30/230）。其中102例患者接受了吉非替尼治疗，在37例具有血浆*EGFR*突变的患者中22例（59.5%）治疗有效，而在65例没有血浆*EGFR*突变的患者中仅15例（23.1%）治疗有效（*P*=0.002），而且血浆*EGFR*突变阳性的患者PFS明显长于没有突变的患者（*P*=0.044）。血浆或组织*EGFR*突变阳性为独立的预后因素（*P*=0.001），说明只要血浆和组织中其一有*EGFR*突变从TKI治疗中获益的可能性就大。

与Bai等^[25]^的研究结果相似，Brevet等^[26]^结合了质谱基因分型检测（mass spectrometry genotyping assay, Sequenom）和突变富集PCR（mutant-enriched PCR, ME-PCR）方法检测血浆DNA中*EGFR*突变，将31例血浆中DNA的*EGFR*突变结果与肿瘤组织DNA进行比较。血浆DNA的*EGFR*突变与原发灶的不一致率为39%（12/31）。厄洛替尼治疗的患者总生存与血浆或肿瘤组织*EGFR*突变密切相关（*P*=0.002），2例只在血浆DNA中有阳性突变的患者也显示很好的疗效和预后。血浆DNA中*EGFR*突变对TKI治疗的疗效具有预测价值。

Zhao等^[27]^比较了93例肺癌组织和配对血浆样本的*EGFR*突变状态。其中3例组织和1例血浆在巢式PCR时未获得目标片段。89例配对血浆和组织的*EGFR*突变一致率为80.9%（72/89）。女性、非鳞癌、晚期患者突变符合率较高，在这些人群中血浆可代替组织标本进行检测。进一步检测证实对鳞癌、早期患者检测血浆基因突变不是一个明智的选择。

Yung等^[28]^测定了35例NSCLC患者靶向治疗前血浆*EGFR*基因突变，具有外显子19缺失和L858R突变的分别有6例（17%）和9例（26%）。与肿瘤样本的测序结果相比较，血浆*EGFR*突变的敏感性和特异性分别为92%和100%。研究发现血浆突变序列的浓度与临床缓解密切相关，在部分缓解或完全缓解的患者中可见到浓度降低，然而在肿瘤进展的患者中可观察到突变持续存在。1例患者在TKI起效后停药，*EGFR*突变在停药后4周再次出现。

上述研究结果提示，血浆和组织的*EGFR*基因突变存在异质性，也具有很大比例的一致性，因此血浆*EGFR*突变状态一定程度上可预测组织的*EGFR*突变状态，而且无论血浆或是组织，只要其一具有突变从TKI治疗中获益的可能性就大。

## 4 化疗前后*EGFR*基因的异质性

这方面的研究较少，以下两项研究结果提示无论是组织中还是血液中*EGFR*基因突变状态均会在化疗后发生一定程度的改变。卓明磊等^[29]^在2010年CSCO会议上报道新辅助化疗前后NSCLC肿瘤组织*EGFR*突变状态的变化。取46例化疗前后肿瘤组织标本进行微切割后提取DNA，采用DHPLC法检测*EGFR*突变状态。全组患者化疗前*EGFR*突变率为43.5%（20/46），化疗后比例降为37%（17/46）。35例患者化疗前后*EGFR*基因型保持不变（22例保持野生型，13例保持突变型），4例由野生型转为突变，7例由突变型转为野生。本组患者34例（78.9%）达到化疗有效，而12例（21.1%）为疾病稳定。多因素分析结果显示化疗方案、周期数、性别、吸烟和病理类型等因素与*EGFR*基因型转换无明显相关。

Wang等^[30]^研究化疗前后血浆*EGFR*突变状态的变化。共有253例患者入组，其中包括203例进展期NSCLC和50例IIIa期NSCLC。血浆样本来源于化疗前（一线治疗前）和化疗后（2个周期化疗后）。*EGFR*的突变用高压液相色谱法测定。203例患者中化疗前的血浆有36.5%（74/203）和化疗后29.6%（60/203）的*EGFR*突变。其中117例在化疗前后保持了原本的*EGFR*基因状态（包括93例野生型和24例突变型），36例由阴性转为阳性，50例由阳性转为阴性。同样的趋势在新辅助化疗中也发现，新辅助化疗前突变率为44%（22/50），新辅助化疗后为36%（18/50），突变率有所下降。以上研究结果提示对于化疗失败拟采用靶向治疗的患者，最好采用即时标本进行*EGFR*突变检测。

综上所述，NSCLC中*EGFR*基因突变存在异质性，不同时间、不同标本、甚至同一标本内都存在*EGFR*基因突变的异质性，这是导致TKI治疗疗效存在差异的原因之一，也提示临床上最好采用即时多点标本的*EGFR*基因检测结果来指导TKI治疗。
